# Aspirin and Statin Use and the Risk of Gallbladder Cancer

**DOI:** 10.3390/cancers13051186

**Published:** 2021-03-09

**Authors:** Kritika Prasai, Sri Harsha Tella, Siddhartha Yadav, Anuhya Kommalapati, Kristin Mara, Mohamed Mady, Mohamed A. Hassan, Nicha Wongjarupong, Natalia Rodriguez-Payan, Mitesh Borad, Tushar Patel, Lewis R. Roberts, Amit Mahipal

**Affiliations:** 1 Division of Gastroenterology and Hepatology, Mayo Clinic, Rochester, MN 55902, USA; prasai.kritika@mayo.edu (K.P.); a01154028@itesm.mx (N.R.-P.); 2 Department of Oncology, Mayo Clinic, Rochester, MN 55902, USA; tella.sri@mayo.edu (S.H.T.); yadav.siddhartha@mayo.edu (S.Y.); kommalapati.anuhya@mayo.edu (A.K.); Mohamed.mady@carle.com (M.M.); 3 Department of Biomedical Statistics and Informatics, Mayo Clinic, Rochester, MN 55902, USA; mara.kristin@mayo.edu; 4 Department of Internal Medicine, University of Pittsburgh Medical Center, Pittsburgh, PA 15260, USA; hassanma@upmc.edu; 5 Department of Medicine, University of Minnesota, Minneapolis, MN 55455, USA; wongj006@umn.edu; 6 Department of Oncology, Mayo Clinic, Scottsdale, AZ 85259, USA; Borad.Mitesh@mayo.edu; 7 Division of Transplantation, Mayo Clinic, Jacksonville, FL 32224, USA; Patel.Tushar@mayo.edu

**Keywords:** aspirin, gallbladder cancer, statin, cancer prevention

## Abstract

**Simple Summary:**

The effects of aspirin on various gastrointestinal cancers have been extensively studied, but the potential protective effect of aspirin and statins on the prevention of gallbladder cancer (GBC) has not been adequately evaluated. The anticancer effect of aspirin has been attributed to direct inhibition of cyclooxygenase (COX)-2. Interestingly, increased expression of COX-2 has been documented in GBC. Hence, we hypothesized that aspirin could potentially have a preventive role in decreasing the risk of GBC. In this study, we demonstrated that the use of aspirin either alone or in combination with statins was associated with a strong reduction in risk of GBC.

**Abstract:**

Aspirin and statin drugs have been associated with reduced risk of several gastrointestinal cancers, but their association with gallbladder cancer (GBC) has not been well established. We evaluated the association of aspirin and statins with the risk of GBC. Patients with GBC managed at Mayo Clinic between 2000 and 2019 were matched 1:2 with a general patient pool by age and sex. Univariable and multivariable logistic regression models were used to assess associations between GBC and aspirin or statin use. The analysis included 795 cases and 1590 controls, with a median age of 67 years. Aspirin or statin use alone or in combination was higher in controls (*p* < 0.001). Univariate analysis showed that the use of aspirin [odds ratio (OR): 0.11; 95%CI: 0.08–0.15] or statins (OR: 0.29; 95%CI: 0.20–0.40) and their combined use (OR: 0.18; 95%CI: 0.13–0.24) was associated with lower risk of GBC. Multivariable analysis revealed that aspirin (OR: 0.12; 95%CI: 0.09–0.16) and combined statins and aspirin (OR: 0.46; 95%CI: 0.31–0.67) were associated with lower risk of GBC. Aspirin alone or in combination with statins is associated with a strongly reduced risk of GBC. Further prospective studies are needed to confirm these results and to elucidate their mechanisms.

## 1. Introduction

Gallbladder cancer (GBC) is a common biliary tract malignancy that accounts for the majority of biliary tract malignancies and has a dismal prognosis [1,2]. The poor survival in GBC is attributed to its biological propensity to metastasis and frequent late presentation at an advanced stage. Given that the only curative approach for GBC is surgical resection of the gallbladder for patients with early-stage disease, finding an effective preventive measure could prove to be of great value. The reduction in cancer risk associated with the use of aspirin and statins has been a matter of interest. Chronic gallbladder inflammation has been established as one of the leading risk factors for GBC [3,4]. Other identified risk factors like cholelithiasis, chronic biliary tract infections, and primary sclerosing cholangitis (PSC) appear to mediate their strong association with GBC through inflammatory mechanisms. Presumably, repeated cycles of mucosal injury and repair through tissue proliferation, the release of inflammatory cytokines, prostaglandins, and growth factors, all predispose gallbladder epithelial cells to oncogenic transformation [5,6].

The effects of aspirin on various gastrointestinal malignancies have been extensively studied [6,7,8,9,10,11], but the potential protective effect of aspirin and statins on the prevention of GBC has not been adequately evaluated. The anticancer effect of aspirin has been attributed to direct inhibition of cyclooxygenase (COX)-2 [12]. As aspirin has been shown to have protective effects on colorectal cancer tumorigenesis, the United States Preventive Services Task Force (USPSTF, https://uspreventiveservicestaskforce.org (accessed on 1 January 2021)) has recommended low-dose aspirin use for the primary prevention of colorectal cancer in select groups of patients. Interestingly, increased expression of COX-2 has been documented in GBC and PSC, but not in the normal bile duct and gallbladder epithelial cells [13]. Hence, we hypothesized that aspirin could potentially have a preventive role in decreasing the risk of GBC.

In addition to aspirin, given the strong association of GBC with dyslipidemia and gallstones, statin medications, which are 3-hydroxy-3-methylglutaryl coenzyme A (HMG-CoA) reductase inhibitors, could possibly play a role in the risk reduction of GBC. Consensus guidelines recommend the use of statins in patients with cardiovascular disease and diabetes mellitus with a higher risk of cardiovascular disease, with the primary aim of cholesterol reduction. In addition, current literature supports gallstone risk reduction with the use of statins [14,15]. Several case-control studies have evaluated the potential role of statins in reducing the risk of breast, lung, colorectal, and prostate cancers, with mixed results [16]. However, the potential role of statins in reducing the risk of GBC is not well established.

To better assess the association of aspirin and statins with the risk of GBC, we conducted a retrospective, observational, case-control study in pathologically confirmed cases of GBC and appropriately matched controls. We also analyzed sex-specific differences for the above association.

## 2. Materials and Methods

### 2.1. Study Design and Participants

Patients with a pathologic diagnosis of GBC between 2000 and 2019 seen at three Mayo Clinic sites in Minnesota, Florida and Arizona, USA were identified using the International Classification of Disease (ICD) codes ICD 10 code (www.cms.gov/icd10 (accessed on 1 January 2021)) C23 or the keywords “gallbladder cancer” or “malignant neoplasm of gallbladder”. Each patient was then matched with two controls in a registry of patients who were evaluated and treated at Mayo Clinic for other health conditions. The cases and controls were matched based on age (age at diagnosis of GBC for cases and age at the time of enrollment for controls) and sex. Information on patient demographics, body mass index, diabetes, hypercholesterolemia, hypertension, hyperthyroidism, hypothyroidism, PSC, inflammatory bowel disease (IBD), and cirrhosis, along with the use of statins and/or aspirin, for both cases and controls, were abstracted from the electronic medical record. The use of aspirin and statins was extracted and confirmed from the documentation of prescription or over-the-counter use.

### 2.2. Statistical Analysis

Descriptive statistics were reported as medians and interquartile ranges for continuous variables, and as frequencies and percentages for categorical variables. Baseline variables were compared between cases and controls using Fisher’s exact test for categorical variables and the Mann–Whitney U test for continuous variables. The primary predictor of interest in the analysis was evaluating the benefits of aspirin and statin medications alone or in combination. Univariable and multivariable conditional logistic regression was used to assess the association between GBC and patient characteristics. Clinical details and laboratory parameters included in the univariable analysis were determined a priori, and variables with a significance level (*p* < 0.05) were included in the multivariable analysis. The data were further stratified by sex to assess and evaluate these associations and the influence of patient sex on the preventive effects of aspirin or statins. As there is a potential possibility of inflammatory bowel disease (IBD) and primary sclerosing cholangitis (PSC) being major confounders, we performed sub-group univariable and multivariable analyses that excluded these variables. Associations are reported using the odds ratio (OR) and 95% confidence interval (CI). Statistical analyses were performed in SAS v9.4 (SAS Institute; Cary, NC, USA) and R statistical software, version 3.4.2 (R Foundation for Statistical Computing, Vienna, Austria). All tests were two-sided, and *p*-values ≤ 0.05 were considered statistically significant.

## 3. Results

A total of 795 patients with pathologically confirmed GBC managed at Mayo Clinic between 2000 and 2019 met the inclusion criteria to be included in the final analysis. The median age at diagnosis was 67 years (range, 27–97 years), and 65% of the study population was female. On 1:2 matching for age and sex, a total of 1590 controls were selected. Table 1 summarizes the demographic characteristics, co-existing clinical conditions, aspirin, and statin use in both the cohorts. In terms of coexisting clinical conditions, as compared to the patients with GBC, the control cohort had a significantly higher percentage of diabetes, hypercholesterolemia, hypertension, and hypothyroidism (*p* < 0.001; Table 1). By contrast, the GBC cohort had significantly higher percentages of patients with IBD and PSC (*p* < 0.001). The prevalence of prescription aspirin use was significantly higher in the control cohort (42.5% vs. 14.2%, *p* < 0.001). Similarly, a significantly higher proportion of the control group was on the combination of aspirin and statins (21.4% vs. 10.8%, *p* < 0.001).

Univariable analysis demonstrated that hypercholesterolemia, hypothyroidism, diabetes, and hypertension were associated with a lower likelihood of GBC (Table 2). A stronger inverse relationship between GBC risk and aspirin (OR: 0.11, 95%CI: 0.08–0.15), statins alone (OR: 0.29, 95%CI: 0.20–0.40), or in combination (OR: 0.18, 95%CI: 0.13–0.24) was observed in the univariable analysis (Table 2). PSC (odds ratio (OR): 6.22, 95%CI: 2.94–13.19) and IBD (OR: 2.06, 95% CI: 1.33–3.20) were strongly associated with an increased risk of GBC.

Multivariable logistic regression analysis including variables that showed statistical significance on univariable analysis revealed that diabetes (*p* = 0.03), hypertension (*p* = 0.01), hypothyroidism (*p* < 0.001), and hypercholesterolemia (*p* < 0.001), use of aspirin alone (OR: 0.12, 95%CI: 0.09–0.16) or in combination with a statin (OR: 0.46, 95%CI: 0.31–0.67) were associated with lower odds of developing GBC (Table 3). There was a trend favoring lower odds of developing GBC with statin use (OR: 0.76, 95%CI: 0.50–1.15). The lack of statistical significance may be attributed to the small percentages of statin users in the cohorts (10% in the GBC cohort, 12% in the control cohort). After matching to all the covariables included, PSC was associated with higher odds of developing GBC (OR: 5.84, 95%CI: 1.91–17.89).

Sub-group stratified analysis based on the sex, showed that the association between aspirin use and lower risk of GBC persisted in both sexes (males: 0.14, 95%CI: 0.09–0.22; females: 0.11, 95%CI: 0.08–0.17) (Table 3). In males, hypercholesterolemia was associated with lower odds of GBC (OR: 0.42, 95%CI: 0.26–0.69) whereas PSC was associated with an increased risk of GBC (OR: 6.54, 95%CI: 1.26–34.03). Similarly, in females, hypercholesterolemia was associated with a lower risk of GBC (OR: 0.27, 95%CI: 0.18–0.39) and PSC was associated with an increased risk of GBC (OR: 5.71, 95%CI: 1.06–30.87) (Table 3). Hypertension (OR: 0.64, 95%CI: 0.48–0.87) and hypothyroidism (OR: 0.52, 95%CI: 0.37–0.73) were associated with a lower risk of GBC in females (Table 3). Aspirin use was associated with a similar risk reduction of GBC in both males and females. However, the combined use of statins and aspirin was associated with a strongly reduced risk of GBC in females (OR: 0.38, 95%CI: 0.23–0.63) but moderate risk reduction in males (OR: 0.56, 95%CI: 0.31–1.01). Further sub-group analysis was performed by excluding the IBD and PSC in both the cohorts and showed that use of aspirin alone or in combination with a statin was associated with decreased risk of GBC (Table 4 and Table 5).

## 4. Discussion

In this case-control study, we demonstrated that the use of aspirin either alone or in combination with statins was associated with lower odds of developing GBC. The study findings are consistent with the previously reported association of aspirin use with a lower risk of other gastrointestinal malignancies found in various geographical regions of the world [7,10,17,18,19,20]. Similarly, a few studies have demonstrated similar risk reductions of GBC with aspirin use. For instance, a Chinese population-based study demonstrated a 63 percent risk reduction of GBC with the use of aspirin [10]. Another study from the United States with 125 GBC cases also demonstrated an association of aspirin use with reduction of GBC risk [20]. It is important to note that risk factors and risk reduction studies in GBC may not be generalized across geographical regions of the world given the variations in etiological factors by region. However, the majority of the risk factors for GBC are thought to trigger chronic inflammation in the gallbladder epithelium, potentially leading to oncogenesis.

In addition to observational clinical studies, multiple experimental studies on aspirin have provided insight into the underlying mechanisms of aspirin in cancer prevention. For instance, the role of COX-2 inhibition by aspirin has been extensively studied in the prevention of colorectal neoplasia [21,22]. COX-2 overexpression has been reported in the tumorigenesis of cholangiocarcinoma and GBC [23,24]. In addition, aspirin has also been shown to exert an onco-protective effect by inhibiting nuclear factor Kappa–B, a transcription factor regulating inflammation and apoptosis [19]. Notably, recent studies have demonstrated the implications of the Wnt signaling pathway in cholangiocarcinoma and GBC tumorigenesis [25]. Interestingly, aspirin was shown to have a regulatory role in the DNA mismatch repair process and Wnt signaling [18,26]. Hence, the use of aspirin can potentially block the effects of chronic inflammation on gallbladder epithelial cells.

For statin alone use, we observed a moderate GBC risk reduction of 0.76, which did not reach statistical significance, likely because of the relatively low percentage of statin use in the cases (10%) and controls (12%). However, when statins are used in combination with aspirin we observed a stronger association with a 54% reduction of GBC risk. Interestingly, our study showed that hypercholesterolemia and use of statins resulted in a lower risk of GBC. One of the possible explanations is that patients who have hypercholesterolemia may be on statins and aspirin resulting in a lowered risk of GBC. Moreover, in addition to aspirin and statins, control of diabetes and hypercholesterolemia may also have been associated with decreased odds of developing gallbladder cancer. Our findings of a potential benefit of statins are consistent with a case-control registry-based analysis from the United Kingdom [27]. It is important to note that the UK study also included patients with cholangiocarcinoma (rather than only GBC as analyzed in the current study). The UK study showed an 11% lower risk of GBC in statin users. Earlier pre-clinical data have shown that statins inhibit tumor cell growth by reducing the synthesis of mevalonate, a precursor for cell cycle regulating metabolic intermediates such as geranyl pyrophosphate, farnesyl-pyrophosphate, and dolichol, which in turn regulate the transcription of oncogenes and cellular DNA synthesis [28]. In addition, statins have also been shown to induce apoptosis of malignant cells and reduce vascular endothelial growth factor (VEGF) production, leading to suppression of tumor growth and metastasis [29,30,31]. Furthermore, statins were demonstrated to have anti-inflammatory effect as they reduce pro-inflammatory interleukins and c-reactive protein (CRP) [32,33].

Our findings of reduced risk of GBC in controls with diabetes, hypercholesterolemia, and hypertension should be interpreted with caution. It is possible that a considerable proportion of patients with these conditions will be on aspirin and statin medications, which might have potentially confounded the results [34]. Another possibility is that there might be a component of detection or surveillance bias as there is a high chance that patients with diabetes mellitus (type 2) and hypercholesterolemia have more frequent liver sonograms for non-alcoholic fatty liver disease (NAFLD) [35]. Nonetheless, statins were shown to alter biliary cholesterol saturation, thereby decreasing gallstone production, which is known to be a risk factor for GBC [36,37]. The current study findings suggest that aspirin and statins seem to have beneficial effects in reducing the risk of GBC after matching these clinical conditions by logistic regression. Future studies are warranted to better elicit the associations of diabetes mellitus, hypercholesterolemia, hypertension, hypothyroidism, and GBC.

The differential effect of the combination of statins and aspirin on the risk reduction of GBC demonstrated in the current study needs a special mention. Given the differential incidence of GBC in females based on the United States Surveillance Epidemiology and End Results (SEER) database, we opted to analyze the effect of aspirin and statins based on the sex [38]. Aspirin use was consistently associated with decreased risk of GBC irrespective of sex. The potential preventive effect of combined use of aspirin and statins was pronounced in females only. Though there was a trend towards potential benefit of statins only in GBC risk reduction in the overall population, there seems to be a confounding factor that is contributing to the wide confidence interval in either sex (Table 3). Though the smaller numbers of patients could possibly explain the lack of statistical significance, future sex-based studies are warranted to further evaluate the potential benefit of statins in reducing GBC risk.

The major strength of the current study is the inclusion of a large number of patients with pathologically confirmed GBC from three geographical regions of the United States. In addition, we obtained controls from the same population pool (patients managed at these facilities for other health conditions) potentially excluding self-selection bias in the analysis. Secondly, we were able to evaluate the possible beneficial effects of aspirin after matching to various important confounders and etiological factors such as body mass index, social habits (smoking, alcohol use), and medical comorbidities such as diabetes, hypercholesterolemia, hypertension, PSC, and inflammatory bowel disease. Third, given the power of the study sample in both the cohorts, we were able to evaluate the beneficial effects of aspirin and statins based on sex.

The study limitations that exist are inherent to retrospective observational analyses. First, due to lack of data on the duration of aspirin and statin use, we were unable to provide an assessment of the effect of duration of use of the drugs with regards to the risk of GBC. Previous studies have demonstrated that duration of use of aspirin can have differential protective effects in colorectal cancer [39]. Such an effect could not be elicited from our analysis of GBC. Future studies are warranted to further evaluate the duration of aspirin therapy in preventing GBC. In addition, as our data were primarily extracted from medical records, we could not evaluate medication compliance. Further, the use of aspirin may not be accurately established due to the availability of the drug as an over the counter medication. If there was a case of non-differential misclassification of aspirin use by case-control status, aspirin may have caused an even more significant risk reduction of GBC than we observed in the present study. Furthermore, we could not exclude any possible drug interactions that aspirin and statins could have with other concurrently administered medications. Lastly, the findings observed in the present study may not be generalized to other geographical regions of the world due to variations in the etiological factors. Hence, the role of chemoprevention of aspirin and statins should be further confirmed by other studies performed in various geographical regions of the world.

## 5. Conclusions

This study demonstrates that the use of aspirin either alone or in combination with statins was associated with a strong reduction in risk of GBC. Additional prospective studies are warranted to confirm this association, to elucidate their underlying mechanisms and the role of duration of aspirin therapy in reducing the risk of GBC.

## Figures and Tables

**Table 1:** Baseline characteristics of cases with gallbladder cancer and controls.

Characteristics	Cases (*N* = 795)	Controls (*N* = 1590)
Age, Median	67	67.5
Female Sex	514 (64.7%)	1028 (64.7%)
Male Sex	281 (35.3%)	562 (35.3%)
**Year of Diagnosis**		
2000–2004	174 (21.9%)	0 (0.0%)
2005–2009	217 (27.3%)	115 (7.2%)
2010–2014	192 (24.2%)	1212 (76.2%)
2015+	212 (26.7%)	263 (16.5%)
BMI, Median (IQR)	27.5 (24.2, 31.9)	29.9 (26.4, 35.0)
Cholelithiasis	383 (48.4%)	0 (0.0%)
Cholecystitis	88 (11.1%)	110 (6.9%)
Diabetes	149 (18.8%)	422 (26.5%)
Hypercholesterolemia	236 (29.8%)	923 (58.6%)
Hypertension	360 (45.4%)	1000 (65.0%)
Hyperthyroidism	6 (0.8%)	6 (0.4%)
Hypothyroidism	110 (13.9%)	364 (22.9%)
PSC	28 (3.5%)	9 (0.6%)
IBD	42 (5.3%)	42 (2.6%)
Cirrhosis	20 (2.5%)	32 (2.0%)
**Smoking**		
No	538 (67.87%)	1247 (78.4%)
Yes	153 (19.2%)	343 (21.6%)
Missing	104 (13.1%)	0 (0.0%)
**Alcohol Abuse**		
No	525 (66.0%)	496 (31.2%)
Yes	23 (2.9%)	1094 (68.8%)
Missing	247 (31.1%)	0 (0.0%)
**Statin**		
Missing	37	0
No	602 (79.4%)	1059 (66.6%)
Yes	156 (20.6%)	531 (33.4%)
**Aspirin**		
Missing	33	3
No	567 (74.4%)	573 (36.1%)
Yes	195 (25.6%)	1014 (63.9%)
**Statin/Aspirin**		
Missing	39	3
Neither	493 (65.2%)	382 (24.1%)
Statin Only	74 (9.8%)	191 (12.0%)
Aspirin Only	107 (14.2%)	675 (42.5%)
Both Statin and Aspirin	82 (10.8%)	339 (21.4%)

**Table 2:** Univariable analysis of the factors evaluated for their association with gallbladder cancer.

Characteristics	Odds Ratio (95% CI)	*p*-Value
BMI (per 1 kg/m^2^)	0.94 (0.92–0.95)	<0.001
Cholecystitis (Yes vs. No)	1.71 (1.26–2.30)	<0.001
Diabetes (Yes vs. No)	0.53 (0.41–0.68)	<0.001
Hypercholesterolemia (Yes vs. No)	0.30 (0.25–0.36)	<0.001
Hypertension (Yes vs. No)	0.44 (0.37–0.53)	<0.001
Hyperthyroidism (Yes vs. No)	2.00 (0.65–6.20)	0.23
Hypothyroidism (Yes vs. No)	0.52 (0.41–0.66)	<0.001
PSC (Yes vs. No)	6.22 (2.94–13.19)	<0.001
IBD (Yes vs. No)	2.06 (1.33–3.20)	0.001
Cirrhosis (Yes vs. No)	1.26 (0.71–2.23)	0.42
Smoking (Yes vs. No)	1.04 (0.84–1.30)	0.71
Alcohol abuse (Yes vs. No)	0.02 (0.01–0.04)	<0.001
Statin (Yes vs. No)	0.53 (0.43–0.65)	<0.001
Aspirin (Yes vs. No)	0.18 (0.15–0.23)	<0.001
**Statin/Aspirin**		
Neither	Reference	
Statin Only	0.29 (0.20–0.40)	<0.001
Aspirin Only	0.11 (0.08–0.15)	<0.001
Both Statin and Aspirin	0.18 (0.13–0.24)	<0.001

**Table 3:** Multivariable analysis of factors evaluated for an association with having gallbladder cancer identified at diagnosis, overall and stratified by gender.

Characteristics	Overall		Male		Female	
	Odds Ratio (95%CI)	*p*-Value	Odds Ratio (95%CI)	*p*-Value	Odds Ratio (95%CI)	*p*-Value
Diabetes						
No	Reference	-	Reference	-	Reference	-
Yes	0.73 (0.55–0.98)	**0.033**	0.79 (0.49–1.27)	0.32	0.71 (0.49–1.02)	0.067
Hypercholesterolemia						
No	Reference	-	Reference	-	Reference	-
Yes	0.31 (0.23–0.42)	**<0.001**	0.42 (0.26–0.69)	**< 0.001**	0.27 (0.18–0.39)	**<0.001**
Hypertension						
No	Reference	-	Reference	-	Reference	-
Yes	0.75 (0.60–0.95)	**0.015**	0.95 (0.65–1.38)	0.78	0.64 (0.48–0.87)	**0.004**
Hypothyroidism						
No	Reference	-	Reference	-	Reference	-
Yes	0.53 (0.39–0.71)	**<0.001**	0.55 (0.28–1.07)	0.078	0.52 (0.37–0.73)	**<0.001**
PSC						
No	Reference	-	Reference	-	Reference	-
Yes	5.84 (1.91–17.89)	**0.002**	6.54 (1.26–34.03)	**0.026**	5.71 (1.06–30.87)	**0.043**
IBD						
No	Reference	-	Reference	-	Reference	-
Yes	1.01 (0.55–1.86)	0.98	1.57 (0.65–3.76)	0.31	0.71 (0.28–1.76)	0.46
Cirrhosis						
No	Reference	-	Reference	-	Reference	-
Yes	0.83 (0.37–1.87)	0.65	2.96 (0.71–12.32)	0.13	0.41 (0.13–1.30)	0.13
Statin/ASA						
Neither	Reference	-	Reference	-	Reference	-
Statin Only	0.76 (0.50–1.15)	0.19	1.03 (0.49–2.19)	0.93	0.67 (0.40–1.12)	0.13
ASA Only	0.12 (0.09–0.16)	**<0.001**	0.14 (0.09–0.22)	**<0.001**	0.11 (0.08–0.17)	**<0.001**
Both Statin and ASA	0.46 (0.31–0.67)	**<0.001**	0.56 (0.31–1.01)	0.053	0.38 (0.23–0.63)	**<0.001**

ASA: Aspirin; IBD: Inflammatory Bowel Disease; PSC: Primary Sclerosing Cholangitis; Significant *p*-values are bolded.

**Table 4:** Univariable analysis excluding potential confounders’ inflammatory bowel disease and primary sclerosing cholangitis.

Characteristics	Odds Ratio (95%CI)	*p*-Value
BMI (per 1 kg/m^2^)	0.94 (0.92–0.95)	<0.001
Cholecystitis (Yes vs. No)	1.74 (1.26–2.41)	<0.001
Diabetes (Yes vs. No)	0.59 (0.45–0.76)	<0.001
Hypercholesterolemia (Yes vs. No)	0.31 (0.26–0.38)	<0.001
Hypertension (Yes vs. No)	0.49 (0.40–0.59)	<0.001
Cirrhosis (Yes vs. No)	0.77 (0.37–1.60)	0.48
Smoking (Yes vs. No)	1.16 (0.91–1.46)	0.23
Alcohol abuse (Yes vs. No)	0.02 (0.01, 0.03)	<0.001
Statin (Yes vs. No)	0.56 (0.45–0.69)	<0.001
Aspirin (Yes vs. No)	0.17 (0.14–0.22)	<0.001
Statin/Aspirin	-	-
Neither	Reference	-
Statin Only	0.31 (0.22–0.44)	<0.001
Aspirin Only	0.10 (0.08–0.14)	<0.001
Both Statin and Aspirin	0.17 (0.13–0.24)	<0.001

**Table 5:** Multivariable analysis of factors (after excluding IBD and PSC) evaluated for an association with having gallbladder cancer overall and stratified by gender.

Characteristics	Overall		Male		Female	
	Odds Ratio (95%CI)	*p*-Value	Odds Ratio (95%CI)	*p*-Value	Odds Ratio (95%CI)	*p*-Value
Diabetes						
No	Reference	-	Reference	-	Reference	-
Yes	0.79 (0.58–1.07)	0.13	0.78 (0.47–1.30)	0.34	0.79 (0.54–1.17)	0.24
Hypercholesterolemia						
No	Reference	-	Reference	-	Reference	-
Yes	0.30 (0.22–0.42)	**<0.001**	0.42 (0.24–0.70)	**0.001**	0.26 (0.17–0.38)	**<0.001**
Hypertension						
No	Reference	-	Reference	-	Reference	-
Yes	0.85 (0.67–1.08)	0.18	1.09 (0.73–1.62)	0.68	0.73 (0.53–0.99)	**0.046**
Cirrhosis						
No	Reference	-	Reference	-	Reference	-
Yes	0.81 (0.33–1.98)	0.64	2.52 (0.59–10.79)	0.21	0.39 (0.10–1.42)	0.15
Statin/ASA						
Neither	Reference	-	Reference	-	Reference	-
Statin Only	0.77 (0.50–1.19)	0.24	1.00 (0.45–2.24)	0.99	0.69 (0.40–1.17)	0.17
ASA Only	0.11 (0.08–0.15)	**<0.001**	0.13 (0.08–0.21)	**<0.001**	0.10 (0.06–0.14)	**<0.001**
Both Statin and ASA	0.42 (0.28–0.63)	**<0.001**	0.55 (0.30–1.02)	0.056	0.33 (0.19–0.57)	**<0.001**

ASA: Aspirin; IBD: Inflammatory Bowel Disease; PSC: Primary Sclerosing Cholangitis; Significant *p*-values are bolded.

## Data Availability

The data presented in this study are available on request from the corresponding author. The data are not publicly available due to institutional restrictions.
